# The Use of Endo-Cellulase and Endo-Xylanase for the Extraction of Apple Pectins as Factors Modifying Their Anticancer Properties and Affecting Their Synergy with the Active Form of Irinotecan

**DOI:** 10.3390/ph15060732

**Published:** 2022-06-09

**Authors:** Jerzy Maksymowicz, Anna Palko-Łabuz, Beata Sobieszczańska, Mateusz Chmielarz, Mirosława Ferens-Sieczkowska, Magdalena Skonieczna, Agnieszka Wikiera, Olga Wesołowska, Kamila Środa-Pomianek

**Affiliations:** 1 Department of Biophysics and Neuroscience, Wroclaw Medical University, 50-367 Wroclaw, Poland; jerzy.maksymowicz@student.umw.edu.pl (J.M.); anna.palko-labuz@umw.edu.pl (A.P.-Ł.); mateusz.chmielarz@student.umw.edu.pl (M.C.); 2 Department of Microbiology, Wroclaw Medical University, 50-367 Wrocław, Poland; beata.sobieszczanska@umw.edu.pl; 3 Department of Chemistry and Immunochemistry, Wroclaw Medical University, 50-367 Wroclaw, Poland; miroslawa.ferens-sieczkowska@umw.edu.pl; 4 Department of Systems Biology and Engineering, The Silesian University of Technology, 44-100 Gliwice, Poland; magdalena.skonieczna@polsl.pl; 5 Biotechnology Centre, Silesian University of Technology, 44-100 Gliwice, Poland; 6 Department of Biotechnology and General Technology of Foods, Faculty of Food Technology, University of Agriculture in Krakow, 31-120 Krakow, Poland; agnieszka.wikiera@urk.edu.pl

**Keywords:** colon cancer, pectin, synergy, SN-38 (irinotecan), apoptosis, inflammation, enzymatic extraction

## Abstract

Pectin constitutes an essential component of dietary fiber. Modified pectins from various sources possess potent anticancer and immunomodulatory activities. In this study, two pectins isolated from apple pomace by *Trichoderma* enzyme treatment, PX (with endo-xylanase) and PCX (with both endo-cellulase and endo-xylanase), were studied in colon cancer cell lines (HCT 116, Caco-2, and HT-29). Both pectins reduced colon cancer cell viability, induced apoptosis, and increased intracellular amounts of reactive oxygen species. Additionally, synergy between pectin and an active form of irinotecan, SN-38, in all aspects mentioned above, was discovered. This drug is a common component of cytotoxic combinations recommended as treatment for colon cancer patients. PX and PCX demonstrated significant anti-inflammatory activity in lipopolysaccharide-stimulated cells. Interaction of apple pectins with galectin-3 and Toll-like Receptor 4 (TLR4) was suggested to be responsible for their anticancer and anti-inflammatory effect. Since PCX was more active than PX in almost all experiments, the role of the enzyme used to obtain the pectin for its biological activity was discussed. It was concluded that co-operation between both enzymes was needed to obtain the molecule of the most beneficial properties. The low molecular mass of PCX together with a high proportion of rhamnogalacturonan I (RG I) regions seemed to be crucial for its superior activity.

## 1. Introduction

Pectins are acidic heteropolysaccharides that may constitute up to 40% of the dry mass of plant cell walls [1] and they are therefore an essential component of dietary fiber consumed by humans. Their structure depends on the plant of origin and the type of plant tissue used as a source, etc. Pectins are complex polymers with highly branched chains. However, some structural regions can always be identified [2]. First, a linear polymer of galacturonic acid that may be methylated and acetylated constitutes the homogalacturonan (HG) region. Next is the rhamnogalacturonan I (RG I) region, whose backbone consists of repeating dimers of galacturonic acid and rhamnose that are further substituted with galactan and arabinan side chains. Finally, there is a highly conserved rhamnogalacturonan II (RG II) region rich in rare saccharides, e.g., L-fucose, apiose, aceric acid, and 2-keto-3-deoxy-D-manno-octulosonic acid. The importance of RG I and RG II presence in pectin molecules has been emphasized for pectin’s biological activity [3,4].

Extracted pectin is mainly used in the food industry as gelling, thickening, emulsifying, and stabilizing agents in a variety of foods [5]. Recently, pectin was also used in the production of edible film coatings [6] as well as drug nanocarriers [7,8]. The recognized biological activities of pectin are very diverse, including the ability to lower serum lipids, cholesterol, and glucose levels, as well as prebiotic activity (reviewed in [9]). Modified pectins were also described as immunomodulating [10], anticancer [11], and proapoptotic agents [12]. These effects are postulated to be mediated via the interaction of pectin with Toll-like Receptor 4 (TLR4) [13] and galectin 3 (Gal-3) [11].

The main commercial sources of pectins are citrus peels and apple pomace [14]. Industrially, pectin is extracted from the plant matrix by temperature treatment (80–100 °C) in acidic conditions (pH 1–3) [15]. Such a procedure, however, results in a reduced amount of RG I and RG II in the product as compared to native pectin. The preparations are enriched in galacturonic acid, while the degree of methylation and the content of neutral sugars is lowered [16]. Therefore, methods in which the enzymatic decomposition of plant material is employed have recently gained more attention [17]. Wikiera et al. [18] have proposed the novel, effective method of enzymatic isolation of apple pectins with the use of fungal endo-cellulase and endo-xylanase. Both enzymes split β–1,4 glycoside bonds within the chains of cellulose and xylan, respectively. Both enzymes co-operate in releasing pectin from cellular walls. The resultant preparation is free from non-pectin sugars. The detailed structure of the obtained pectins was previously described [18], and their properties and biological activity were partially characterized [19]. Shortly, the enzymatically prepared apple pectins had very high molecular masses and contained a significant amount of branched RG I and RG II regions and were highly methylated and rich in neutral sugars (such as arabinose, rhamnose, and galactose), characteristic of pectins in muro.

In our previous study [20], the promising anticancer activity of apple pectin PC, obtained with the use of endo-cellulase, was demonstrated. Its anticancer properties in colon cancer cell line HCT 116 were shown to be superior to commercially available modified citrus pectin, PectaSol. Additionally, the existence of synergy between PC and the active form of irinotecan (SN-38) was proven. In the present work, two other enzymatically extracted apple pectins were studied. PX was obtained with the use of endo-xylanase, and PCX by the treatment of apple pomace by both endo-cellulase and endo-xylanase. Anticancer properties of the apple pectins were studied in the panel of colon cancer cell lines (HCT 116, Caco-2, and HT-29). Colon cancer cell lines have been chosen as a research model since colon cells are likely to come in direct contact with pectin as plant-origin or pectin-containing foods are consumed. Both pectins were demonstrated to reduce colon cancer cell viability, induce apoptosis, and increase the amount of reactive oxygen species (ROS) in cancer cells. Additionally, the synergy between pectin and SN-38 in all aspects mentioned above was discovered. Moreover, apple pectins had an anti-inflammatory effect in colon cancer cells, possibly via the interaction with TLR4. Since PCX turned out to be more active than PX in almost all experiments, the role of the type of enzyme used to obtain the pectin for its biological activity was discussed.

## 2. Results

### 2.1. Cytotoxicity Assay

#### 2.1.1. Cytotoxicity of Pectins in Colon Cancer Cells

**Figure 1:**
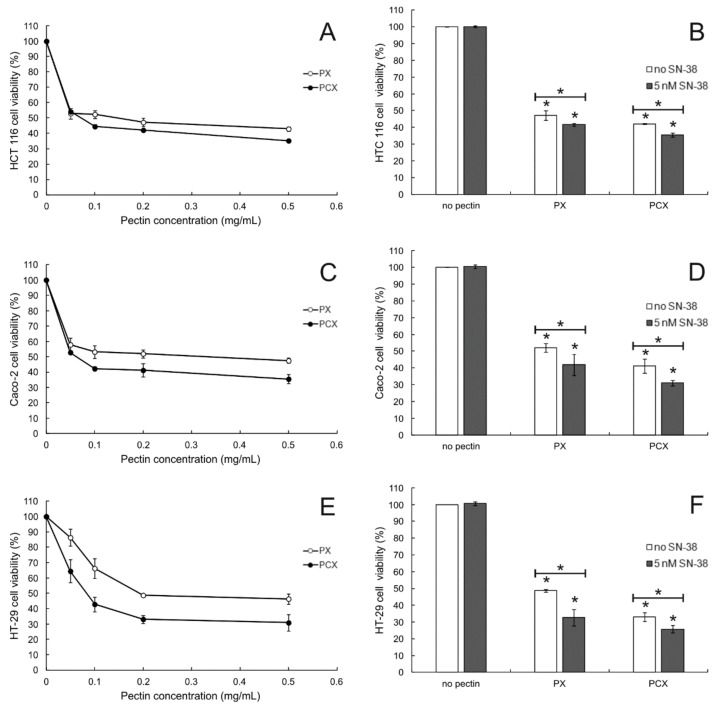
MTT cytotoxicity assay of pectins in HCT 116 (**A**), Caco-2 (**C**), HT-29 cells (**E**), and 0.2 mg/mL pectins in combination with SN-38 ((**B**,**D**,**F**) for HCT 116, Caco-2, and HT-29 cells, respectively). Incubation time was 48 h. The means of the three experiments ± SD are presented (* *p* < 0.05). Statistical significance was checked between the studied probes and controls (no pectin) and between probes containing only pectin and pectin combined with SN-38.

The anticancer activity of apple pectins was studied in three colon cancer cell lines: HCT 116, Caco-2, and HT-29. Colorectal cancers are molecularly heterogeneous and can be divided into clinically relevant subtypes associated with patient prognosis and treatment response [21]. HT-29, HCT-116 and Caco-2 are model colon cancer cell lines commonly employed in research. They differ in gender origin and mutation status [22]. Additionally, HT2-9 is colon adenocarcinoma, while HCT-116 and Caco-2 are colon carcinoma and primary colon tumor, respectively. The results of MTT assay proved that both PX and PCX were cytotoxic to all the cell lines studied (Figure 1, left column). However, even at the highest concentration tested (0.5 mg/mL), pectins were not able to kill more than ca. 60% of cells. In all experimental settings, PCX was more cytotoxic to colon cancer cells than PX (*p* < 0.05 for all concentrations tested). IC_50_ values of the studied pectins are presented in Table 1. Except for HCT 116 cells, the IC_50_ values recorded for PCX were ca. twice lower than the ones obtained for PX. The SRB assay experiments brought virtually the same results (Figure S1). Additionally, the toxicity of the studied pectins towards the non-cancer cells was studied. Both pectins at the concentration of 0.2 mg/mL reduced the viability of FHC cells (fetal human colon epithelium) by about 20% (Figure S2).

#### 2.1.2. Cytotoxic Effect of Pectins Combined with SN-38

Figure 1 (right column) also presents the effect of anticancer drug SN-38 (an active form of irinotecan) on the cytotoxic potential of PX and PCX. It was visible that the combination of each apple pectin (at 0.2 mg/mL) with the non-toxic concentration of SN-38 (5 nM) resulted in the enhancement of the cytotoxic effect in all colon cancer cell lines. Both pectins exhibited significant cytotoxicity when applied alone, but the co-treatment of cancer cells with low concentration of SN-38 increased cytotoxicity even more.

**Figure 2:**
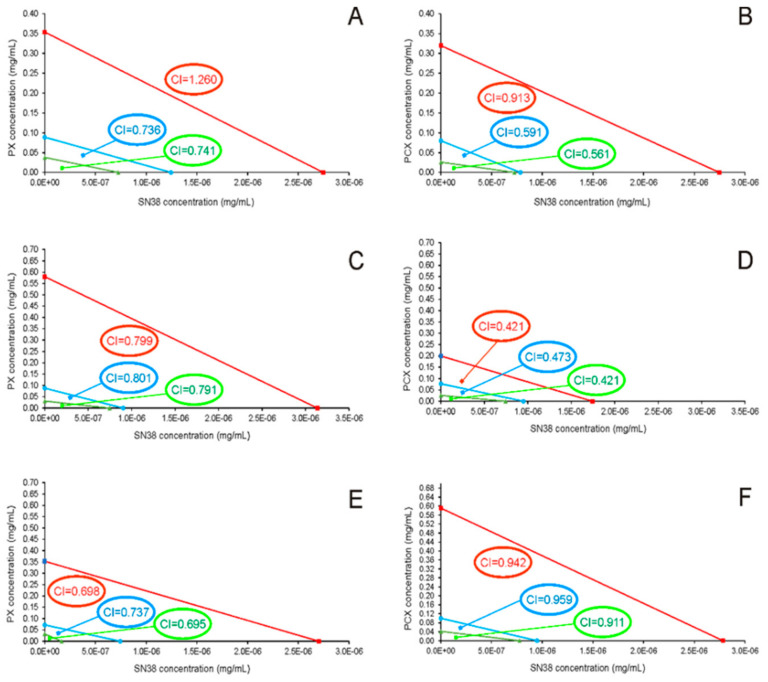
Isobolograms for the interaction of PX (**A**,**C**,**E**) and PCX (**B**,**D**,**F**) with SN-38 in HCT-116 (**A**,**B**), Caco-2 (**C**,**D**), and HT-29 cells (**E**,**F**). The isobolograms were constructed by connecting the IC_30_ (triangles), IC_50_ (circles), and IC_70_ (squares) values of pectins with the appropriate IC values of SN-38. Lines indicate the theoretical lines of additivity.

**Figure 3:**
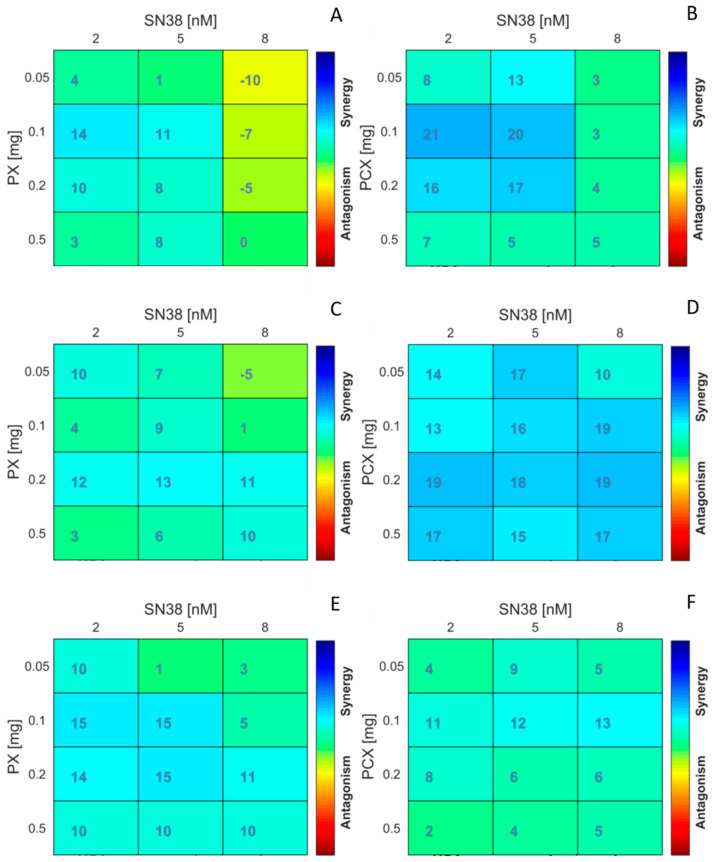
Synergism of apple pectins PX (**A**,**C**,**E**) and PCX (**B**,**D**,**F**) with anticancer drug SN-38 in colon cancer cells HCT 116 (**A**,**B**), Caco-2 (**C**,**D**), and HT-29 (**E**,**F**). The matrices show Combenefit analysis of the combinations. MTT cell viability data after 48 h of incubation (arithmetically averaged data of three independent experiments) were used for the analysis. Synergy score: less than −5—the interaction between two drugs is likely to be antagonistic; from −5 to 5—the interaction is likely to be additive; larger than 5—the interaction is likely to be synergistic.

This prompted us to perform isobolographic analysis to detect the existence of putative synergy between the apple pectins and the anticancer drug. For pure compounds and their combinations, dose and effect data were obtained from the MTT assay. The analysis via CompuSyn software based on the model of Chou and Martin [23] yielded combination index values (CI) for PX:SN-38 and PCX:SN-38 mixtures (Table 2). For almost all the combinations, CI values were clearly below 1, suggesting synergy between the apple pectins and SN-38. As judged by CI values, in HCT 116 and Caco-2 cells the synergy between PCX and SN-38 was more potent than in case of PX. Surprisingly, this effect was reverted in HT-29 cells. The obtained isobolograms are presented in Figure 2. Additional analysis performed using Combenefit software [24] (Figure 3) corroborated the synergy between the studied substances. Matrix format plots present synergy scores calculated according to the Highest Single Agent (HSA) model [25], and the level of antagonism or synergism is represented by a color scale bar. As can be noticed in both HCT 116 and Caco-2 cells, but not in HT-29 cells, PCX exhibited stronger synergy with SN-38 than PX. Additionally, it can be noticed that the synergistic effect predominantly occurred in low concentrations of anticancer drug, i.e., below its IC_50_ value (that ranged between 7.5 and 8 nM depending on colon cancer cell line).

### 2.2. Cell Cycle Analysis

**Figure 4:**
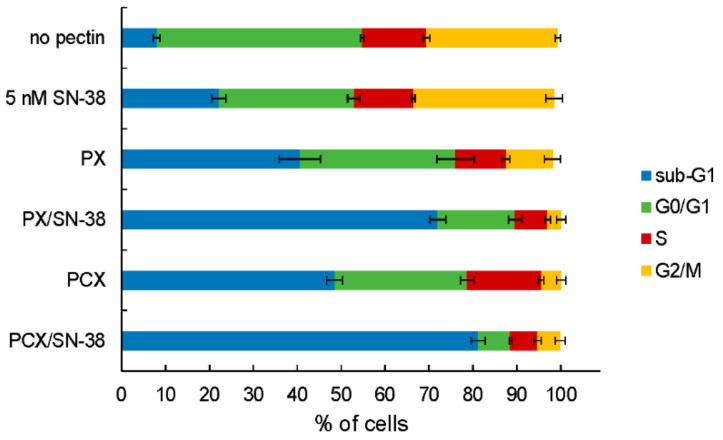
Cell cycle-dependent DNA content in HT-29 cells treated with 0.2 mg/mL of pectins and/or 5 nM SN-38 for 48 h. Sub-G_1_ population—dead cells, G_0_/G_1_—mononuclear cells, S—DNA replication, G_2_/M—mitosis. The means of three experiments ± SD are presented.

**Figure 5:**
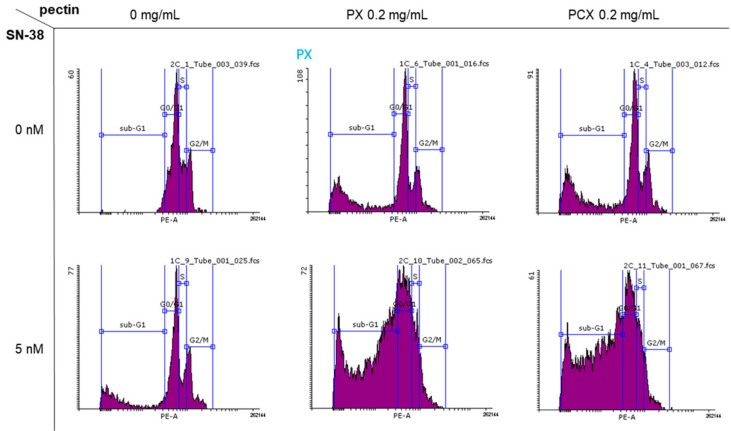
Typical histograms of DNA content (stained with PI) in HT-29 cells treated with 0.2 mg/mL of pectins and/or 5 nM SN-38 for 48 h. Sub-G_1_ population—dead cells, G_0_/G_1_—mononuclear cells, S—DNA replication, G_2_/M—mitosis.

Flow cytometric analysis of cellular DNA content was employed to investigate the influence of pectins on the cell cycle of colon cancer cells. Figure 4 presents the proportion of HT-29 cells in different cycle phases and Figure 5 presents the exemplary histograms. It can be noticed that the treatment of cells with 0.2 mg/mL of both PX and PCX caused the significant (* *p* < 0.05 as compared to the untreated control) increase of the sub-G_1_ fraction of HT-29 cells with a concomitant decrease in other fractions, especially G_0_/G_1_. Cells in the sub-G_1_ phase are predominantly dead cells, i.e., necrotic or late apoptotic, while G_0_/G_1_ phase represents living mononuclear cells. The addition of 5 nM of SN-38 also increased the share of sub-G_1_ cells, however, this was to a lesser extent than pectins themselves. Most interestingly, the combination of pectin and SN-38 resulted in a robust increase in the number of HT-29 cells in the sub-G_1_ phase. This pointed to the mutual enhancement of activity between the anticancer drug and apple pectins. Similar results were obtained when the influence of the studied pectins was investigated in HCT 116 cells and Caco-2 cells (Figure S3).

### 2.3. Apoptosis Detection

**Figure 6:**
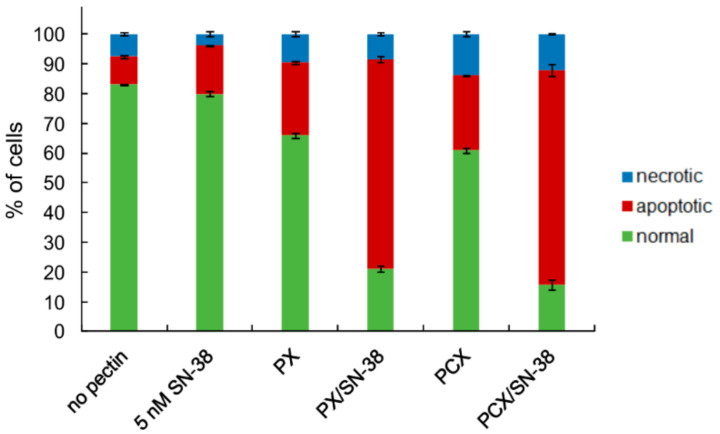
The proportion of normal, apoptotic, and necrotic cell populations as recorded by Annexin-V apoptosis assay in HT-29 cells treated with pectins (0.2 mg/mL) and/or SN-38 for 48 h. The means of three experiments ± SD are presented. Cells were recognized as viable (Annexin-V and PI negative), apoptotic (Annexin-V positive and PI negative), and necrotic (Annexin-V and PI positive) based on the measurement of cell-associated fluorescence of FITC-Annexin-V conjugate and PI.

The finding that the treatment of colon cancer cells with pectins affected their cell cycle, causing the increase of dead cells share, prompted us to perform a more detailed analysis of the putative mechanism of cellular death caused by the studied compounds. The results of cytometric experiments on the detection of apoptotic cells are presented in Figure 6. It is clearly visible that the treatment of HT-29 cells by both pectins resulted in an increase in the number of apoptotic cells as compared to the cells not treated with the studied pectins (* *p* < 0.05). SN-38 applied at 5 nM concentration also slightly increased the population of apoptotic cells. However, the most pronounced effect was observed when the pectins were used in combination with the anticancer drug. The proportion of apoptotic cells raised dramatically. It is worth noticing that in all cases, the factor responsible for reducing the normal cell population was the significant increase of the number of apoptotic cells, while the population of necrotic cells increased only slightly. Similar results were also obtained for HCT 116 and Caco-2 cells (Figure S4).

Additionally, the ability of apple pectins to affect the activity of caspase-3 was studied. This proteolytic enzyme is associated with the execution phase of the apoptotic cascade. As shown in Table 3, both apple pectins caused the activation of caspase-3 in all studied cell lines. At the same concentration (0.2 mg/mL), PCX turned out to be a more potent caspase-3 activator than PX. Anticancer drug SN-38, at 5 nM, virtually did not affect the enzyme’s activity. However, the combination of the drug with pectins produced the significant activation of apoptosis-associated caspase. The results of the described above experiments pointed out that apple pectins, especially in combination with SN-38, manifested potent proapoptotic activity in colon cancer cells.

### 2.4. Oxidative Stress Detection

**Figure 7:**
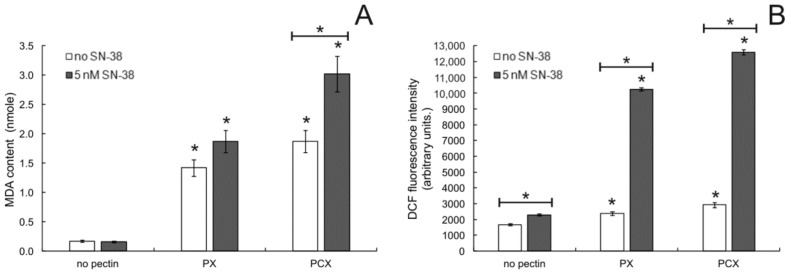
Lipid peroxidation (**A**) and ROS level (**B**) in HT-29 cells treated with 0.2 mg/mL pectins and/or 5 nM SN-38 for 48 h. The means of three experiments ± SD are presented (* *p* < 0.05). Statistical significance was checked between the studied probes and controls (no pectin) as well as between probes containing only pectin and pectin combined with SN-38.

To better understand the mechanism by which apple pectins induced apoptosis in colon cancer cells, the effect of pectins on the degree of oxidative stress in cells was studied. The lipid peroxidation status of cells was determined by measuring the amount of peroxidation product MDA (malondialdehyde) in cells treated by pectins. As shown in Figure 7A, both PX and PCX, when used at 0.2 mg/mL increased the level of lipid peroxidation in HT-29 cells. The active form of irinotecan, SN-38, when applied at low concentration (5 nM), had virtually no effect on the studied parameter. On the other hand, when HT-29 cells were treated with SN-38 in combination with pectins, a further increase of lipid peroxidation was recorded, more pronounced in the case of PCX than PX. Nearly the same results were also obtained in HCT 116 and Caco-2 colon cancer cells (Figure S5).

Next, the generation of ROS in colon cancer cells in the presence of apple pectins was quantified. The results of the assay in which intracellular ROS were detected with the use of fluorescent probe DCF are presented in Figure 7B. Both studied pectins (at 0.2 mg/mL) significantly increased ROS levels in HT-29 cells, and the degree of this increase was comparable to the effect caused by SN-38 at 5 nM. The combination of pectins with the low concentration of anticancer drug resulted in a dramatic rise in the amount of ROS generated in colon cancer cells. This effect was slightly stronger for the PCX:SN-38 combination than for PX:SN-38. Similar observations were also made in two other colon cancer cell lines studied (Figure S5); however, in HCT 116 cells, PX was more effective in augmenting the ROS-generating potency of SN-38 than PCX.

### 2.5. Modulation of Inflammation by Pectins

**Figure 8:**
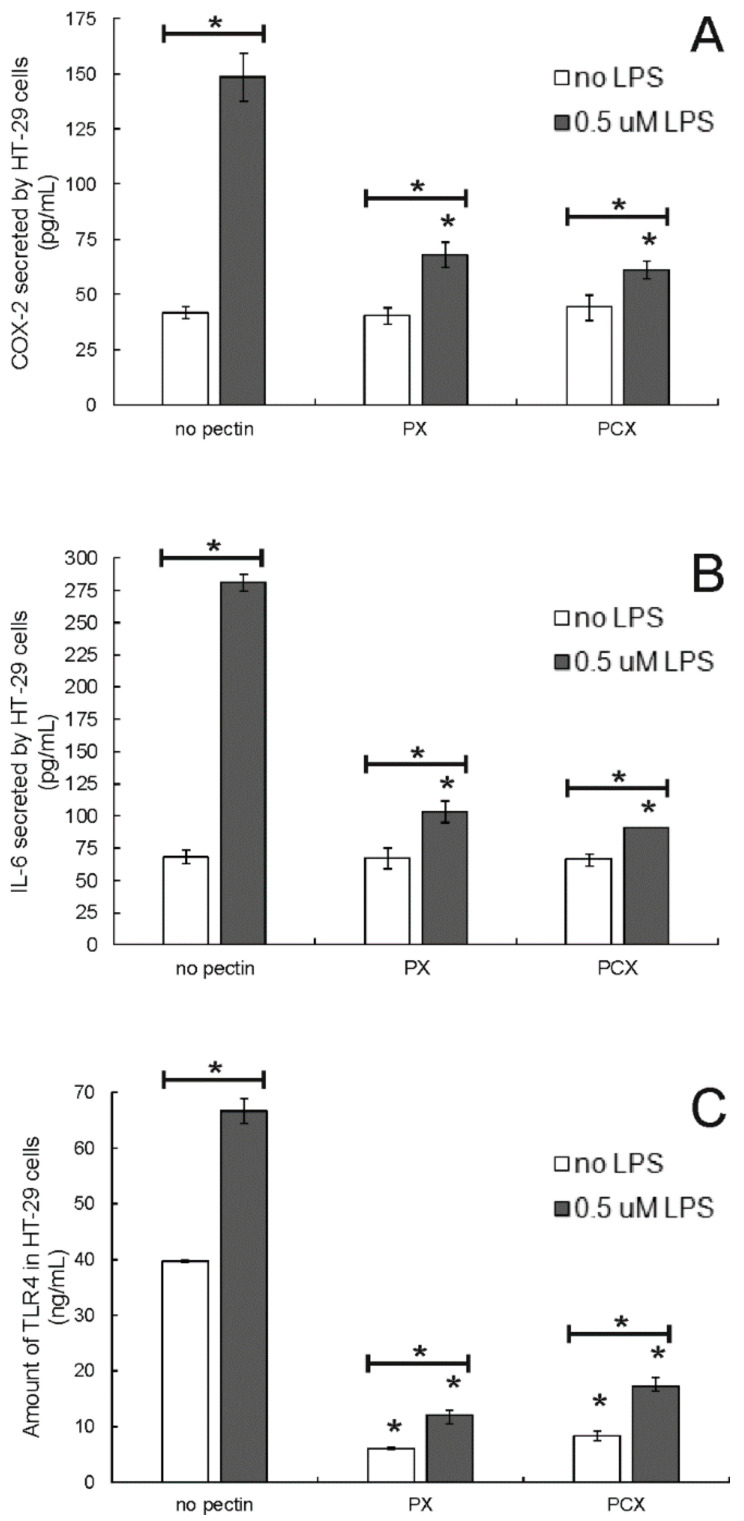
Amount of COX-2 (**A**), IL-6 (**B**) and TLR4 (**C**) in HT-29 cells treated with 0.2 mg/mL pectins and/or 0.5 μM LPS. Cells were pretreated with LPS for 24 h and then incubated with pectins for 48 h. The means of three experiments ± SD are presented (* *p* < 0.05). Statistical significance was checked between the studied probes and controls (no pectin) as well as between probes containing only pectin and pectin combined with LPS.

Colon cancer cells were pretreated by lipopolysaccharide (at 0.5 μM) for 24 h to induce inflammation. The ability of apple pectins to modulate the cellular inflammation process was investigated in a model system prepared in such a way. As shown in Figure 8, the treatment of HT-29 cells with LPS resulted in a significant increase in both cyclooxygenase 2 (COX-2) and interleukin 6 (IL-6) amounts. Pectins (applied at 0.2 mg/mL) did not affect the level of both inflammation markers in the cells not stimulated by LPS. On the other hand, apple pectins were able to strongly reduce the level of COX-2 (Figure 8A) and IL-6 (Figure 8B) secreted by LPS-pretreated colon cancer cells, which pointed to their anti-inflammatory potency. Similar results were obtained in two other colon cancer cell lines: HCT 116 and Caco-2 (Figure S6).

TLR4 plays the role of main cellular receptor for LPS, and its activation leads to the activation of cytokine production. As shown in Figure 8C, the pretreatment of HT-29 cells by LPS resulted in a significant increase in the amount of TLR4. The studied apple pectins, PX and PCX, caused a significant decrease in the amount of this protein in colon cancer cells that had been both stimulated and not stimulated by LPS.

### 2.6. Galectin-3 Detection

**Figure 9:**
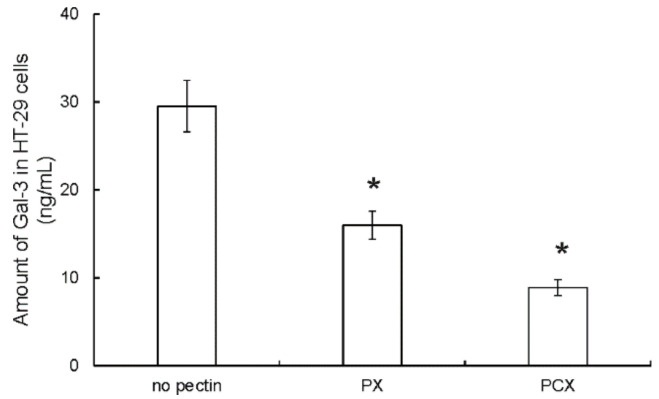
Amount of Gal-3 in HT-29 cells treated with 0.2 mg/mL pectins for 48 h. The means of three experiments ± SD are presented (* *p* < 0.05). Statistical significance was checked between the studied probes and controls (no pectin).

Since both studied pectins induced apoptosis in colon cancer cells, it was also checked whether they could affect the cellular amount of galectin-3 (Gal-3). Gal-3 possesses many functions. Among others, it is engaged in apoptosis regulation. Investigation of Gal-3 by means of ELISA assay showed that the treatment of HT-29 cells by PX or PCX (at 0.2 mg/mL) resulted in a pronounced drop in the amount of Gal-3 detected in cell lysates (Figure 9). Additionally, PCX was demonstrated to affect the Gal-3 amount more strongly than PX. Similar observations were made in HCT 116 and Caco-2 cells (Figure S7).

### 2.7. E. coli Adherence to Cancer Cells

**Figure 10:**
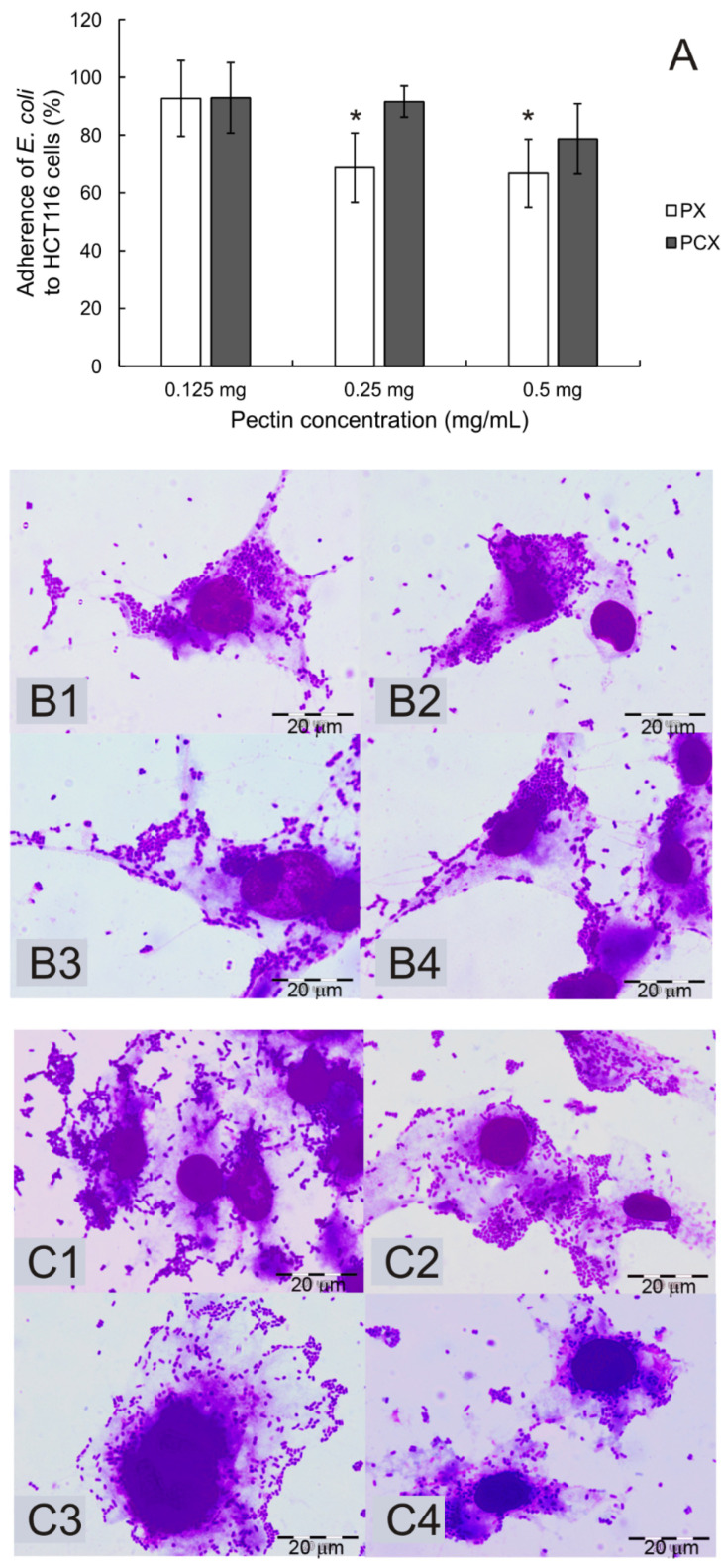
Adherence of the *E. coli* LF82 strain to HCT 116 cells (**A**) in the presence of pectins during a 2 h incubation period. The means of three experiments ± SD are presented. Statistical significance (* *p* < 0.05) was assessed between the experimental groups and controls (no pectin), which were assumed to be 100%. Representative images of bacteria adhering to untreated HCT 116 cells (**B1**,**C1**) and cells treated with PX (**B**) or PCX (**C**) at concentrations of 0.125 mg/mL (**B2**,**C2**), 0.25 mg/mL (**B3**,**C3**), and 0.5 mg/mL (**B4**,**C4**). Wright–Giemsa stain, 100× magnification.

Since the interaction of all compounds with colonic cells might be modulated by the local microbiota, the ability of the pectins to interfere with the adherence of *Escherichia coli* was tested in HCT 116 cells (Figure 10). The adherent-invasive strain of bacteria (LF82) isolated from a patient with Crohn’s disease was used in experiments. PX inhibited the adherence of LF82 to epithelial cells by ca. 30% (* *p* < 0.05). PCX was less active in this respect. It exerted a slight inhibition on *E. coli* adherence at the highest concentration used, however, statistical significance was not reached.

## 3. Discussion

The anticancer activity of enzymatically extracted apple pectins, PX, and PCX, was demonstrated in a panel of colon cancer cell lines. Both studied pectins were cytotoxic to HCT 116, Caco-2, and HT-29 cells. IC_50_ values recorded for PCX in HT-29 and Caco-2 cells were almost twice lower than those for PX. In HCT 116 cells, IC_50_ values for both pectins were comparable but PCX was still more cytotoxic than PX. Anticancer properties of PX and PCX have been previously studied in adenocarcinoma and melanoma cells [19]. It was found that colon cancer cells were more sensitive to pectins than melanoma cells. In contrast, the viability of normal mouse fibroblasts was not affected by pectins in the studied concentrations. Recently, cellulase-extracted apple pectin, PC, was demonstrated to reduce the viability of colon cancer cells [20]. Pectins isolated from other plant sources, such as potato [26] and ginseng [27], were previously shown to also be anticancer agents in human colorectal adenocarcinoma HT-29 cells. It is worth noticing that the superiority of the anticancer activity of pectin preparations rich in RG I regions over the ones depleted in these regions was reported [12,26]. On the other hand, pectin preparations from jaboticaba fruit [28] and papaya [29], which are rich in galacturonic acid (that suggested high content of HG regions), were demonstrated to be selectively toxic to HCT 116 and HT-29 cells as compared to the preparations with higher contents of neutral sugars.

The cytotoxic properties of the apple pectins towards colon cancer cells were also studied in the presence of a low concentration of the active form of irinotecan (SN-38). The drug was used at the concentration of 5 nM, which was practically non-toxic to none of the studied cell lines. When SN-38 and apple pectins were applied together, the studied compounds exerted a much more pronounced cytotoxic effect on all types of the colon cancer cells. Isobolographic analysis clearly demonstrated the existence of synergy between the apple pectins and SN-38. For PX, the severity of the observed synergistic effect was similar in all three cell lines studied. On the other hand, PCX exhibited more potent synergy with SN-38 in HCT 116 and Caco-2 cells than in HT-29 cells. Therefore, it was concluded that the processes that resulted in synergy were dependent on the type of cancer cells. Together with our previous observation [20], this is the first demonstration of the synergistic effect between apple pectin and the anticancer drug. Previously, only modified citrus pectin was shown to exhibit synergy with paclitaxel in ovarian cancer cells [30], and doxorubicin in prostate cancer cells [31].

The observation of the cytotoxic effect of enzymatically extracted apple pectins on colon cancer cells was supplemented by the study of their effect on cell cycle and apoptosis induction. In all three cell lines, the presence of pectins resulted in an increase in the number of cells in the sub-G_1_ phase of the cell cycle (necrotic and late apoptotic cells) as well as the induction of apoptosis (documented as the elevated number of cells that had externalized phosphatidylserine and also as the activation of caspase-3). When the binary combination of the apple pectin and SN-38 was applied to colon cancer cells, the enhancement of the effect of pectins both on the cell cycle and apoptosis induction was recorded. Proapoptotic properties of various pectin preparations in colon cancer cells have been previously reported. Pectins obtained from sugar beetroot and sweet potato were found to induce apoptosis in HT-29 cells [12,32]. The HG-rich ginseng pectin caused HT-29 cell cycle arrest in the G_2_/M phase and induced apoptosis accompanied by the activation of caspase-3 [27]. Enzymatically isolated apple pectin, PC, induced apoptosis in human colorectal carcinoma cells HCT 116 [20], and enzymatically prepared low molecular mass citrus pectin reduced viability, induced apoptosis, and caused cell cycle arrest in the S phase in liver cancer HepG2 cells [33]. Finally, it was shown that fish oil- and citrus pectin-enriched diet protected rats from chemically or radiation-induced colon cancer by upregulating apoptosis in colonic mucosa [34].

Next, the ability of the apple pectins to affect the level of lipid peroxidation and intracellular ROS was investigated. It was found that the studied pectins significantly increased the levels of both markers of oxidative stress in colon cancer cells. Additionally, in the presence of SN-38, the effect of pectins was highly augmented. This suggested that pectins displayed pro-oxidative properties in the experimental settings. Such properties might complement the cytotoxic potential of the apple pectins towards colon cancer cells since the enhancement of ROS production might lead to cellular death via triggering apoptosis. This observation is in apparent contrast with the numerous reports on the antioxidative activity of various pectin preparations (reviewed in [35]), including apple pectin [19]. However, it should be kept in mind that the results obtained in simple laboratory models are not necessarily likely to translate into cellular conditions. The ROS-increasing potency of various pectins has been previously observed. Prostate cancer cells exhibited increased radiosensitivity in the presence of modified citrus pectin, which was associated with the elevated ROS production [36]. Salehi et al. [37] described that citrus and apple pectins induced apoptosis in breast cancer cells through the dysregulation of permeability and subsequent destruction of the mitochondrial membrane that allowed for the excessive ROS release. The increase in ROS production was also recorded in human glioblastoma cells treated with pectins from *Campomanesia xanthocarpa* [38]. Finally, enzymatically extracted apple pectin, PC, has been observed to increase ROS production in colon cancer HCT 116 cells [20]. Pectins are believed to interact with Gal-3, which was shown to influence ROS production, NADPH oxidase enzyme expression, and redox signalling [39]. It can therefore be supposed that the increase of cellular amount of ROS observed in the presence of various pectins might be mediated via their interaction with Gal-3.

The chronic inflammatory process is nowadays believed to lie at the root of the development of many progressive diseases, including cancer. Since the anti-inflammatory activity of pectins has been widely observed (for a review see [40]), it was decided to study apple pectins in this respect. Bacterial LPS was used to induce inflammation. Its application resulted in the significant increase in COX-2 and IL-6 levels in all colon cancer cell lines tested. PX and PCX strongly decreased the amounts of both inflammation markers while not affecting their levels in non-stimulated cells. The ability of pectins to mitigate the expression or activity of various inflammation markers in variable LPS-stimulated cell types has been widely recognized. For example, citrus pectin reduced the expression of COX-2 and inducible nitric oxide synthase (iNOS) [41], and raspberry pectin diminished NO and IL-6 production [42] in stimulated macrophages. Expression of IL-6 was also diminished in LPS-treated murine microglia in the presence of RG I-rich pectin from lotus germs [43]. When LPS-stimulated colon cancer cells (HT-29 and SW-620) were treated by apple oligogalactan [44] or modified apple polysaccharides [45], the expression of COX-2, as well as other inflammation markers, was significantly lowered. Apple pectin, PC, also decreased the amounts of COX-2 and IL-6 in colon cancer cells HCT 116 pretreated with LPS [20]. Ginseng polysaccharides were demonstrated to alleviate colitis symptoms in rats, accompanied by the downregulation of inflammatory cytokines (IL-1β, IL-2, IL-6, and IL-17) [46]. Additionally, antibiotic-associated diarrhea in mice was relieved by the polysaccharide from the rhizome of *Dioscorea opposite*, which also attenuated the expression of IL-1β and IL-6 in colon tissues [47].

Toll-like receptors (TLRs) play a key role in pro-inflammatory signaling networks due to their ability to recognize a variety of pathogen-associated products (e.g., lipids, proteins, lipoproteins, and nucleic acids) and subsequent triggering of the production of inflammatory cytokines, co-stimulatory molecules, interferons, and chemokines [48]. TLR4 is known to be the cellular receptor for LPS produced by Gram-negative bacteria. Since the direct interaction of pectins with TLR4 has been proposed to constitute the mechanism of the immunomodulatory activity of pectins [10], the ability of enzymatically extracted apple pectins to bind to TLR4 was studied by means of an ELISA assay. A measurable amount of TLR4 was detected only in HT-29 cells but not in HCT 116 and Caco-2 cells. The stimulation of colon cancer cells did not change the situation. This is in agreement with the analysis of Suzuki et al. [49], who detected the presence of TLR4 protein in the cytoplasmic fraction of HT-29 and Colo205 cells but not in HCT 116 and Caco-2. This suggested that some other receptors had to be associated with the LPS-responsiveness observed in the two latter cell lines. For instance, TLR2 has also been postulated to serve as the receptor for LPS [50]. As shown by the results of the experiments, both PX and PCX significantly decreased the amount of TLR4 accessible to antibodies in HT-29 cells that were both stimulated and non-stimulated by LPS. Therefore, it was concluded that both studied pectins bound to the TLR4 receptor. The effect of pectins on TLR4 function and expression has already been observed in several laboratory settings, including the experimental models in which the conditions of inflammation-associated diseases were mimicked in animals. Modified citrus pectin downregulated the expression of TLR4 in rats with myocardial fibrosis [51], and an oligogalactan from apple pectin effectively reduced the elevated levels of TLR4 in a mouse model of colitis-associated colon cancer [52]. Moreover, modified apple polysaccharide was shown to suppress TLR4 expression and thus the TLR4-signalling pathway in colon cancer cells [45]. The authors pointed to the competition between LPS and apple polysaccharide for binding to TLR4. Park et al. [53] demonstrated that the anti-TLR4 antibody blocked the immunomodulatory effect of RG I-type polysaccharide from citrus in macrophages. Additionally, the colocalization of RG II with TLR4 in bone marrow dendritic cells was shown [54].

Another essential regulatory protein with which pectins are known to interact is galectin-3. Gal-3 belongs to the lectin family and specifically recognizes β-galactosides (see [55] for a review). Due to the specific binding to various protein targets, Gal-3 mediates numerous biological processes associated with cellular growth, cancer transformation, invasion, and metastasis. The changes in its expression have been reported to occur in many cancer and pre-cancerous conditions. Intracellular Gal-3 is believed to act as an anti-apoptotic factor, so its overexpression is likely to make cancer cells partially resistant to apoptosis. In the present study, an ELISA assay was employed to investigate the interaction of the apple pectins with Gal-3. In all colon cancer cell lines, the presence of pectins caused a decrease in the amount of Gal-3 detected by the antibodies. Additionally, PCX seemed to affect Gal-3 to a larger extent than PX. Therefore, it was concluded that the studied pectins directly interacted with Gal-3, which might be responsible for their observed antiproliferative and proapoptotic activity. Since the first observation of Nangia-Makker et al. [56]—that modified citrus pectin can bind to Gal-3 and act as its competitive inhibitor, many studies have reported the direct interaction between these molecules. Binding between various types of pectins or polysaccharides with Gal-3 has been detected via nuclear magnetic resonance [57,58], surface plasmon resonance [59,60], and atomic force microscopy [61], as well as fluorescence microscopy and flow cytometry [60]. The inhibition of the Gal-3-mediated hemagglutination of erythrocytes by pectins has also been observed [29,62,63].

Interaction of all dietary compounds with colonic cells might be modulated by the local microbiota. Therefore, it was checked whether enzymatically extracted apple pectins could affect the adherence of *Escherichia coli* to colon cancer cells. As both colon cancer and Crohn’s disease have been associated with the local inflammation of the colon, an adherent-invasive strain of bacteria (LF82) isolated from a Crohn’s disease patient was used in the experiments. Reduced adhesion of LF82 to cancerous epithelial cells in the presence of PX demonstrated the interference of pectin with *E. coli* adherence. LF82 adhesion is determined by mannose-sensitive type 1 fimbriae that bind to mannose receptors on the surface of intestinal epithelial cells. Pectins containing, among others, mannose groups, can interfere with the adhesion of LF82 to the intestinal epithelium that was also shown in our previous study on endo-cellulase-extracted pectin [20].

In summary, we have clearly shown that the apple pectins PX and PCX exhibited antiproliferative, proapoptotic, and anti-inflammatory activities in colon cancer cells. Moreover, both pectins exhibited synergy with SN-38 in reducing the viability of cancer cells. As with the molecular mechanism of anticancer and anti-inflammatory properties of the pectins, the putative interaction with Gal-3 and TLR4 was suggested, respectively. Interestingly, PCX turned out to be a more active antiproliferative agent than PX, while their anti-inflammatory activity was comparable. Moreover, PCX also exhibited a stronger synergy with SN-38. This prompted us to attempt to analyze the features of three different enzymatically extracted apple pectins that might be important for their anticancer activity. PX was obtained by apple pomace treatment with endo-xylanase, PC with endo-cellulase, and PCX with both enzymes [18]. The anticancer properties of PC have been already studied in HCT 116 cells [20], and it turned out to be more potent than commercially available modified citrus pectin, PectaSol, but less active than PCX. The composition of all three apple pectins has been already characterized [18]. Compared with the standard acid-extracted pectin, all had high molecular masses, were highly methylated, and contained a significant amount of branched RG I and RG II regions. PX was characterized by the highest mass (ca. 900 kDa), the lowest content of galacturonic acid, and the highest content of neutral sugars. PC was intermediate in all discussed aspects, while PCX had the lowest mass (ca. 420 kDa) and the smallest amount of neutral sugars. However, it must be stressed that PCX still contained more branched regions than commercially produced apple pectin. When one looks at literature reports discussing the parameters crucial for the antiproliferative activity of pectins and/or their ability to interact with Gal-3 (which is believed to be the primary mechanism responsible for pectin’s anticancer potency), a complicated picture emerges. First, relatively low molecular mass was associated with the beneficial activity, as various types of modified pectins were more potent anticancer agents than naturally large pectins [29,64,65]. The presence of RG I regions has been stressed to be necessary for antiproliferative effect of pectins by many authors [12,26,65]. Also, the content of various types of arabinogalactan side chains was pointed out as an important factor [12,66,67]. In particular, galactans rich in terminal β-galactosides are frequently considered to be “pharmacophores” for Gal-3 binding [67,68]. On the other hand, some reports suggested that the presence of non-esterified HG regions (that translated to high content of glacturonic acid) was necessary for the anticancer activity of pectins [28,29,69]. This apparent discrepancy was explained by realizing that both the RG I/HG backbone and the galactan/arabinan side chains were engaged in the anticancer activity of pectins [12]. The requirement for the correct RG I/HG ratio and the co-operation of both regions was also noticed [61]. Zhang et al. [69] proposed that the interaction of HG with RG I forced the correct spatial arrangement of the pectin molecule in which binding epitopes for Gal-3 (containing galactose and arabinose residues) were exposed. The right proportion between HG and RG I regions and their three-dimensional arrangement seemed also to be the factor explaining the observed differences in activity between the three apple pectins. Although PX contained the highest proportion of branched regions, it was not the most active anticancer compound. The obstacle might be its high molecular mass; such a large molecule was likely to adopt a conformation in which arabinogalactan side chains would be covered and protected from the binding with Gal-3. PCX, which was characterized by the lowest molecular mass, exhibited the superior antiproliferative activity that led us to the conclusion that the co-operation between both endo-xylanase and endo-cellulase was needed to obtain the molecule of the most beneficial properties. An interesting observation was recently made in the study on pectins isolated from papaya fruit at different stages of ripening process [65]. It was shown that the activity of natural plant enzymes, such as polygalacturonases and β-galactosidases, produced during ripening and causing the solubilization of cell walls and fruit softening, resulted in the pectin preparation with potent antiproliferative activity towards colon cancer cells HCT 116 and HT-29 and high potency for Gal-3 binding. This suggests that using natural, plant, fungal or microbial enzymes may constitute a promising direction in the development of pectin extraction methods.

## 4. Materials and Methods

### 4.1. Chemicals

SN-38 (7-ethyl-10-hydroxy-camptothecin), an active metabolite of irinotecan, was a product of Sigma-Aldrich (Poznan, Poland). Stock solution of SN-38 was prepared in dimethyl sulfoxide (DMSO) and stored at −20 °C. Stock solutions of pectins were prepared at a concentration of 10 mg/mL in double-distilled water. Stock solutions were diluted in a culture medium just before the experiments.

### 4.2. Pectin Isolation

Macromolecular apple pectin was extracted from dried apple pomace using endo-cellulase (endo-β-1,4-glucanase, EC 3.2.1.4, Sigma-Aldrich, Poznan, Poland) and endo-xylanase (endo-β-1,4-xylanase, EC 3.2.1.8, Sigma-Aldrich, Poznan, Poland) produced by filamentous fungus *Trichoderma viride*. Isolation was performed with 50 U of enzyme per 1 g of pomace, at pH 5.0, in 40 °C, for 10 h, according to the method described by Wikiera et al. [18]. Briefly, dried and ground apple was treated by the appropriate enzyme for 10 h in conditions of pH 5.0 at 40 °C with constant shaking. After the extraction, the samples were cooled down to 20 °C and centrifuged. Next, cold 96% ethanol (4 °C) was added to the supernatants to the final concentration of 70%. The precipitated pectin was collected by centrifugation, washed with 70% ethanol, centrifuged again and the pellets were dried at 60 °C for 24 h until the constant weight was achieved. The resulting pectins were ground to a particle passing through a 60-mesh screen (0.251 mm). The subsequent characterization of the obtained pectins yielded the following parameters [18]. Molecular mass of PX was 899 kDa, galacturonic acid content was 61.1%, degree of methylation was 73.4%, and neutral sugars content was 29.8%. PCX molecules were smaller (molecular mass of 419 kDa), less methylated (56.1%) contained more galacturonic acid (74.7%), and less neutral sugars (17.9%).

### 4.3. Cell Culture

The studies were performed on human colon cancer cell lines: HT-29, HCT 116, Caco-2 and FHC, obtained from ATCC collection. The cells were cultured in medium DMEM-F12 (PAA) supplemented with 1% antibiotics (10,000 μg/mL streptomycin and 10,000 units/mL penicillin; Sigma-Aldrich (Poznan, Poland) and fetal bovine serum (FBS; Eurx). The concentration of FBS in medium was 10% for HCT 116. HT-29, and FHC cells, and 20% for Caco-2 cells. Moreover, DMEM-F12 for Caco-2 cells contained non-essential amino acid solution (1×) (Sigma-Aldrich, Poznan, Poland). The medium for FHC cell line was additionally supplemented with 0.005 mg/mL transferrin, 100 ng/mL hydrocortisone, 20 ng/mL human recombinant EGF. The cells were cultivated at 37 °C in a humidified atmosphere with 5% CO_2_, under standard conditions. The medium was changed twice a week. Confluent cultures were passaged using 0.25% trypsin. All experimental procedures were carried out in the log-phase of cell growth.

### 4.4. Cell Viability Assay

Cells were seeded on 96-well plates (150,000 cells/mL) 24 h before the experiment. Then, the medium was removed, and a fresh medium containing studied pectins (PX or PCX) in the concentration of 0.05; 0.1; 0.2; or 0.5 mg/mL was added. In the experiments in which the combination of pectin and SN-38 was used, the concentration of PX or PCX was 0.2 mg/mL, whereas the concentration of the drug was 5 nM. The appropriate control containing DMSO in fresh medium was also prepared. The plates were incubated for 48 h at 37 °C in a humidified atmosphere with 5% CO_2_. Cell viability was assessed using MTT (3-(4,5-dimethylthiazol-2-yl)-2,5-diphenyltetrazolium bromide) method, as described previously [20]. In general, the assay is based on the reduction of a yellow tetrazolium salt (MTT) to purple formazan crystals by metabolically active cells. The absorbance of the final product was read at 570 nm using a microplate reader. Survival rate was expressed as the percentage of cell survival and calculated from the ratio (A_570_ of treated cells/A_570_ of control cells) × 100%. The experiments were repeated three times.

The sulforhodamine B (SRB) assay [70] was modified and used for estimation of cytotoxic properties of the studied compounds. Cells were seeded (60,000/well) onto 96-well plates with the compounds: pectins or SN-38, used alone or in combination in the appropriate concentrations. DMSO concentration in the samples did not exceed 0.5%. The plates were incubated for 48 h at 37 °C. Subsequently, 50% cold trichloroacetic acid was added. After incubation for 60 min at 4 °C, cells were washed with tap water and stained with 0.4% sulforhodamine B (Sigma-Aldrich, Poznan, Poland) for 30 min at room temperature. The dye was removed by washing under 1% acetic acid. Then, the plates were dried, and 10 mmol/L Tris (pH = 10.5) was added to each well. Protein concentration was estimated from the measurements of absorbance at 491 nm. Survival rate was expressed as the percentage of cell survival and calculated from the ratio (A_491_ of treated cells/A_491_ of control cells) × 100%. The experiments were repeated three times.

### 4.5. Isobolographic Analysis

The combination index (CI) was calculated using CompuSyn software (ComboSyn Inc., Paramus, NJ, USA) according to the classic median-effect Equation (1), as was previously described by [23].

(1) $$\mathrm{CI}=\frac{{\left(D\right)}_{1}}{{\left(\mathrm{Dx}\right)}_{1}}+\frac{{\left(D\right)}_{2}}{{\left(\mathrm{Dx}\right)}_{2}}$$

In the Equation (1), (Dx)_1_ is the dose of drug 1 alone that inhibits a system by x%, (Dx)_2_ is the dose of drug 2 alone that inhibits a system by x%, and (D)_1_ + (D)_2_ are the doses of drug 1 and 2 in combination that also inhibit a system by x%. CI values below 1 represent synergism, CI value equal to 1 indicates additive effect (i.e., no interaction), and CI values above 1 point to antagonism.

Combenefit is a software tool that enables the visualization, analysis, and quantification of substance combination effects [24]. Data taken from MTT assays of pectin:drug combinations were processed using classical Highest Single Agent (HSA) synergy model [25].

### 4.6. Flow Cytometry

In order to study cell cycle, an apoptosis and intracellular level of reactive oxygen species (ROS) flow cytometry method was implemented. The cells (150,000 cells/mL) were seeded onto 12-well plates 24 h before the experiment. Then, the medium was removed, and a fresh medium containing pectin (PX or PCX) at the concentration of 0.2 mg/mL with or without SN-38 at the concentration of 5 nM was added. The appropriate control containing DMSO in fresh medium was also prepared. The cells were incubated for the next 48 h at 37 °C. Further procedures was carried out as described previously [20]. Briefly, the cells were harvested and centrifuged (235× *g*, 3 min, room temperature). The apoptosis was investigated using an Annexin-V apoptosis assay (BioLegend, San Diego, CA, USA). To study cell cycle, the cells were fixed with hypotonic buffer containing propidium iodide (PI) at 100 µg/mL in PBS; 5 mg/L of citric acid; 1:9 Triton-X solution; RNase 100 µg/mL in PBS (Sigma-Aldrich, Poznan, Poland). The level of ROS was measured using a cell-permeable non-fluorescent probe, 2’,7’-dichlorofluorescin diacetate (DCFH-DA, Sigma-Aldrich, Poznan, Poland), which undergoes hydrolysis by cellular esterases into its polar form DCFH. Due to the intracellular ROS and other peroxides oxidation process takes place and DCFH turns into highly fluorescent compound 2’,7’-dichlorofluorescein (DCF). Cytometric analyses were performed using an Aria III flow cytometer (Becton Dickinson, Franklin Lakes, NJ, USA) with FITC configuration (488 nm excitation; emission: LP mirror 503, BP filter 530/30) or with PE configuration (547 nm excitation; emission: 585 nm), and at least 10,000 cells were counted. All experiments were performed in triplicate.

### 4.7. Caspase-3 Activity

The activity of caspase-3 was analyzed using a commercially available kit (GenScript Biotech, Leiden, the Netherlands), according to the manufacturer’s protocol. Cells were seeded (800,000 cells/well) onto a 6-well plate in 2 mL of medium and incubated for 24 h at 37 °C. Then, the medium was removed, and a fresh medium containing pectin (PX or PCX) at concentration of 0.2 mg/mL with or without SN-38 at 5 nM was added. Control wells contained the cancer cells in fresh medium with DMSO. The cells were incubated for the next 48 h (at 37 °C), then scraped and centrifuged (2000× *g*, 5 min, 25 °C). A further procedure was carried out as described previously [20]. In general, the assay based on the spectrophotometric detection of chromophore p-nitroaniline (pNA) (at 405 nm) released by caspase-3 from the labeled substrate DEVD-pNA. The relative increase of caspase-3 activity was determined by calculating the ratio of the absorbance of pNA in the studied sample (treated with the compound) to the control (with no compound). Experiments were performed in triplicate.

### 4.8. MDA Content

The level of malondialdehyde (MDA), a product of lipid peroxidation which is a biological marker of oxidative stress, was analyzed using Lipid Peroxidation (MDA) Assay Kit (Abcam/Symbios, Straszyn, Poland), according to the manufacturer’s instructions with minor modifications. Cells were seeded (800,000 cells/well) onto a 6-well plate in 2 mL of medium and incubated for 24 h at 37 °C. Then, the medium was removed, and a fresh medium containing pectin (PX or PCX) at concentration of 0.2 mg/mL with or without SN-38 at 5 nM was added. The cells were incubated for next 48 h (37 °C). After incubation, the cells were harvested and washed with PBS. The pellet of cells was suspended in Lysis Solution containing MDA Lysis Buffer and butylated hydroxytoluene (BHT). The cells were homogenized on ice and then centrifuged (13,000× *g*, 10 min) to remove insoluble material. In order to generate MDA-TBA adducts, supernatant was mixed with tiobarbituric acid (TBA) and incubated for 60 min at 95 °C. Then, the samples were cooled to room temperature and added into a 96-well microplate for analysis. A microplate reader was used to measure the absorbance at 532 nm. In order to determine MDA concentration in the samples, a standard curve was used.

### 4.9. Enzyme-Linked Immunosorbent Assay (ELISA)

The enzyme-linked immunosorbent assay (ELISA) method was used to detect and quantify cyclooxygenase-2 (COX-2), interleukin-6 (IL-6), toll-like receptor 4 (TLR4), and Gal-3. First, the cells (HT-29, HCT 116 or Caco-2) were seeded in 96-well plates (150,000 cells/mL). In order to stimulate immune response lipopolysaccharide (LPS; trinitrophenol-lipopolysaccharide from *E. coli* O111:B4, Sigma-Aldrich, Poznan, Poland) in the concentration of 5 μM was added to each well 24 h before the experiment. This step was omitted in case of Gal-3 assay. Next, the cells were treated by the studied pectins at the concentration of 0.2 mg/mL for 48 h. IL-6, COX-2, TLR4, and Gal-3 were detected in cancer cells culture lysates using IL-6 Human ELISA Kit (ThermoFisher Scientific, Waltham, MA, USA), Human COX-2 ELISA Kit (Sigma-Aldrich, Poznan, Poland), Human TLR-4 ELISA Kit (Sigma-Aldrich, Poznan, Poland), and Human Galectin-3 ELISA Kit (Sigma-Aldrich, Poznan, Poland), respectively. The studies were performed according to the manufacturer’s instructions. All experiments on the detection and quantification of selected proteins were repeated three times.

### 4.10. Adherence Assay

The adherence of bacteria cells to colon cancer cells in the presence of pectins, PX or PCX, was investigated as previously described [20]. An adherent-invasive strain of *E. coli* (LF82) isolated from a patient with Crohn’s disease was kindly provided by Dr. Arlette Darfeuille-Michaud (Université d’Auvergne, France). HCT 116 cells were infected with *E. coli* and incubated with PX or PCX in concentrations of 0.125, 0.25, and 0.5 mg/mL. Cells untreated with pectins and infected with bacteria constituted a positive control. After 2 h of incubation (37 °C, 5% CO_2_), the cells were washed with PBS and lysed with 0.1% Triton X-100. Serial dilutions of bacterial lysates were plated onto nutrient agar and incubated overnight at 37 °C to count bacterial colonies (CFU). The experiment was repeated three times in duplicate. The results are presented as the percentage of *E. coli* adhering to untreated cells, with negative control established to be 100%.

Adhesion images were taken under a light microscope after Wright–Giemsa staining. HCT 116 cells were cultured on slides in the wells of a 24-well plate. After 2 h of adherence experimentation, HCT 116 cells were washed and fixed with formaldehyde in the concentration of 4% (10 min, room temperature). Then, formaldehyde was washed off and the cells were stained with Wright–Giemsa for 30 min. The dye was rinsed off, and the slides were dried and inspected under a microscope.

### 4.11. Statistical Analysis

The values of measured parameters were presented as the means ± standard deviation (SD) from three independent experiments. The statistical significance was determined by Student *t*-test (0.05 as threshold value) with the use of Statistica 10 software.

## 5. Conclusions

Summing up, the antiproliferative, proapoptotic, and anti-inflammatory properties of enzymatically extracted apple pectins were demonstrated in colon cancer cells. Additionally, the synergy between pectin and SN-38 was discovered. The interaction of the apple pectins with Gal-3 and TLR4 was the molecular mechanism for their anticancer and anti-inflammatory effect. It was concluded that the relatively low molecular mass of PCX together with the relatively high proportion of RG I regions gave this pectin superior anticancer activity.

## Figures and Tables

**Table 1:** Cytotoxic potential of the apple pectins against colon cancer cells.

Cell Line	PX	PCX
	IC_50_ (mg/mL)	IC_50_ (mg/mL)
HCT 116	0.167 ± 0.011	0.116 ± 0.009
Caco-2	0.208 ± 0.012	0.090 ± 0.008
HT-29	0.190 ± 0.012	0.080 ± 0.007

IC_50_ values (concentrations needed to produce 50% reduction in cell number) were determined by Combenefit software.

**Table 2:** Combination of apple pectins with SN-38 against colon cancer cell growth.

Cell Line	Concentration (mg/mL)			Ratio	Combination Index (CI)
	SN-38	PX	PCX		
	0.78 × 10^−6^	0.1	-	128,200:1	0.629
	1.96 × 10^−6^	0.2	-	102,000:1	0.863
HCT 116	3.14 × 10^−6^	0.5	-	157,730:1	1.010
				
	0.78 × 10^−6^	-	0.1	128,200:1	0.543
	1.96 × 10^−6^	-	0.2	102,000:1	0.561
	3.14 × 10^−6^	-	0.5	157,730:1	0.905
	0.78 × 10^−6^	0.1	-	128,200:1	0.895
	1.96 × 10^−6^	0.2	-	102,000:1	0.791
	3.14 × 10^−6^	0.5	-	157,730:1	0.821
Caco-2					
	0.78 × 10^−6^	-	0.1	128,200:1	0.599
	1.96 × 10^−6^	-	0.2	102,000:1	0.424
	3.14 × 10^−6^	-	0.5	157,730:1	0.489
	0.78 × 10^−6^	0.1	-	128,200:1	0.725
	1.96 × 10^−6^	0.2	-	102,000:1	0.726
	3.14 × 10^−6^	0.5	-	157,730:1	0.735
HT-29					
	0.78 × 10^−6^	-	0.1	128,200:1	0.822
	1.96 × 10^−6^	-	0.2	102,000:1	0.941
	3.14 × 10^−6^	-	0.5	157,730:1	0.956

Dose and effect data were obtained from the MTT assay (mean values of three experiments) and analyzed by CompuSyn software. CI values were calculated by CompuSyn software. CI = 1 indicates additive effect, CI < 1—synergism, and CI > 1—antagonism.

**Table 3:** Relative caspase-3 activity in colon cancer cells treated with pectins (at 0.2 mg/mL) and pectins in combination with SN-38 (at 5 nM) for 48 h.

Cell Line	Pectin (mg/mL)	Relative Caspase-3 Activity	
			+5 nM SN-38
HCT 116	0	1.00 ± 0.00	1.06 ± 0.06
	0.2 PX	1.40 ± 0.06 *	2.36 ± 0.04 * ^§^
	0.2 PCX	1.47 ± 0.03 *	2.43 ± 0.02 * ^§^
Caco-2	0	1.00 ± 0.00	1.08 ± 0.01
	0.2 PX	1.59 ± 0.05 *	2.40 ± 0.00 * ^§^
	0.2 PCX	1.69 ± 0.02 *	2.63 ± 0.02 * ^§^
HT-29	0	1.00 ± 0.00	1.18 ± 0.19
	0.2 PX	1.59 ± 0.01 *	2.55 ± 0.08 * ^§^
	0.2 PCX	1.67 ± 0.03 *	2.76 ± 0.25 *

The means of three experiments ± SD are presented (* *p* < 0.05). Statistical significance was checked between the studied probes and controls (no pectin), and between the probes containing only pectin and pectin together with SN-38 (^§^ *p* < 0.05).

## Data Availability

Data is contained within the article and supplementary material.

## Supplementary Materials

The following are available online at https://www.mdpi.com/article/10.3390/ph15060732/s1. Figure S1: SRB cytotoxicity assay of pectins in HCT 116 (**A**), Caco-2 (**C**), HT-29 cells (**E**), and 0.2 mg/mL pectins in combination with SN-38 ((**B**,**D**,**F**) for HCT 116, Caco-2, and HT-29 cells, respectively), incubation time 48 h. Figure S2: MTT cytotoxicity assay of pectins at 0.2 mg/mL in FHC cells. Incubation time was 48 h. Figure S3: Cell cycle-dependent DNA content in HCT 116 (**A**) and Caco-2 (**B**), cells treated with 0.2 mg/mL of pectins and/or SN-38 for 48 h. Figure S4: The proportion of normal, apoptotic, and necrotic cell populations as recorded by annexin V apoptosis assay in HCT 116 (**A**) and Caco-2 (**B**) cells treated with pectins (0.2 mg/mL) and/or SN-38 for 48 h. Figure S5: Lipid peroxidation (**A**,**C**) and ROS level (**B**,**D**) in HCT 116 (**A**,**B**) and Caco-2 (**C**,**D**) cells treated with 0.2 mg/mL pectins and/or 5 nM SN-38 for 48 h. Figure S6: Amount of COX-2 (**A**,**C**) and IL-6 (**B**,**D**) in HCT 116 (**A**,**B**) and Caco-2 (**C**,**D**) cells treated with 0.2 mg/mL pectins and/or 0.5 μM LPS (cells were pretreated with LPS for 24 h and then incubated with pectins for 48 h). Figure S7: Amount of Gal-3 in HCT 116 (**A**) and Caco-2 (**B**) cells treated with 0.2 mg/mL pectins for 48 h.
