# Correction: Maksymowicz et al. The Use of Endo-Cellulase and Endo-Xylanase for the Extraction of Apple Pectins as Factors Modifying Their Anticancer Properties and Affecting Their Synergy with the Active Form of Irinotecan. *Pharmaceuticals* 2022, *15*, 732

**DOI:** 10.3390/ph18020152

**Published:** 2025-01-24

**Authors:** Jerzy Maksymowicz, Anna Palko-Łabuz, Beata Sobieszczańska, Mateusz Chmielarz, Mirosława Ferens-Sieczkowska, Magdalena Skonieczna, Agnieszka Wikiera, Olga Wesołowska, Kamila Środa-Pomianek

**Affiliations:** 1 Department of Biophysics and Neuroscience, Wroclaw Medical University, 50-367 Wroclaw, Poland; 2 Department of Microbiology, Wroclaw Medical University, 50-367 Wrocław, Poland; 3 Department of Chemistry and Immunochemistry, Wroclaw Medical University, 50-367 Wroclaw, Poland; miroslawa.ferens-sieczkowska@umw.edu.pl; 4 Department of Systems Biology and Engineering, The Silesian University of Technology, 44-100 Gliwice, Poland; magdalena.skonieczna@polsl.pl; 5 Biotechnology Centre, Silesian University of Technology, 44-100 Gliwice, Poland; 6 Department of Biotechnology and General Technology of Foods, Faculty of Food Technology, University of Agriculture in Krakow, 31-120 Krakow, Poland

## Error in Figure

In the original publication [1], there was a mistake in Figure 10 as published. By mistake, we doubled panels B2–B4 and presented them as panels C2–C4 which of course should not be identical to panel B. This error was not intentional; however, we realized that it may lead to confusion among readers. The corrected Figure 10 appears below. The authors state that the scientific conclusions are unaffected. This correction was approved by the Academic Editor. The original publication has also been updated.

## Figures and Tables

**Figure 10:**
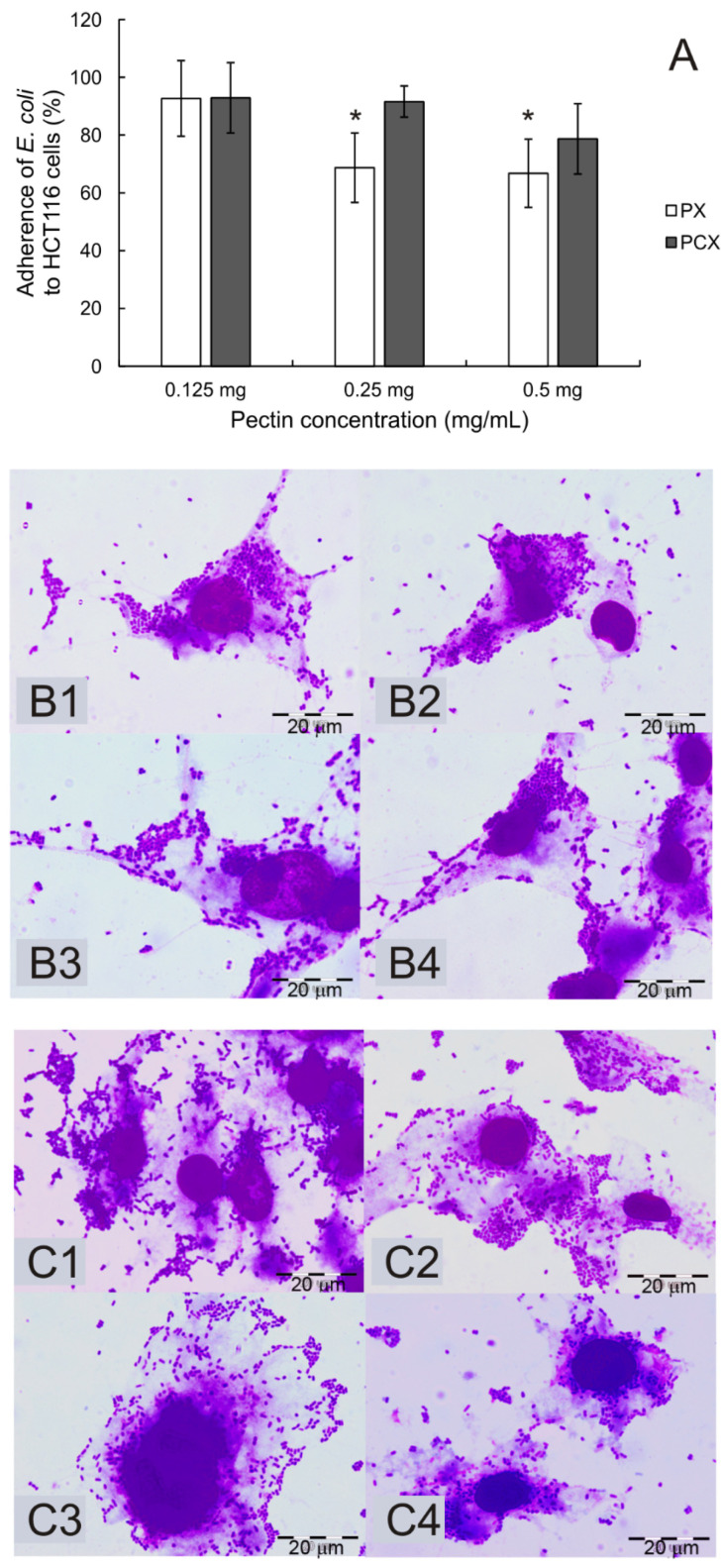
Adherence of the *E. coli* LF82 strain to HCT 116 cells (**A**) in the presence of pectins during a 2 h incubation period. The means of three experiments ± SD are presented. Statistical significance (* *p* < 0.05) was assessed between the experimental groups and controls (no pectin), which were assumed to be 100%. Representative images of bacteria adhering to untreated HCT 116 cells (**B1**,**C1**) and cells treated with PX (**B**) or PCX (**C**) at concentrations of 0.125 mg/mL (**B2**,**C2**), 0.25 mg/mL (**B3**,**C3**), and 0.5 mg/mL (**B4**,**C4**). Wright–Giemsa stain, 100× magnification.
