# Supplementary material for: The Use of Endo-Cellulase and Endo-Xylanase for the Extraction of Apple Pectins as Factors Modifying Their Anticancer Properties and Affecting Their Synergy with the Active Form of Irinotecan
Source: Pharmaceuticals (Basel). 2022 Jun 9;15(6):732. doi: 10.3390/ph15060732 (PMC9229824; doi:10.3390/ph15060732)
Supplement: Supplementary file 1 [file pharmaceuticals-15-00732-s001.zip › pharmaceuticals-1727562-supplementary.pdf]

Supplementary figures:

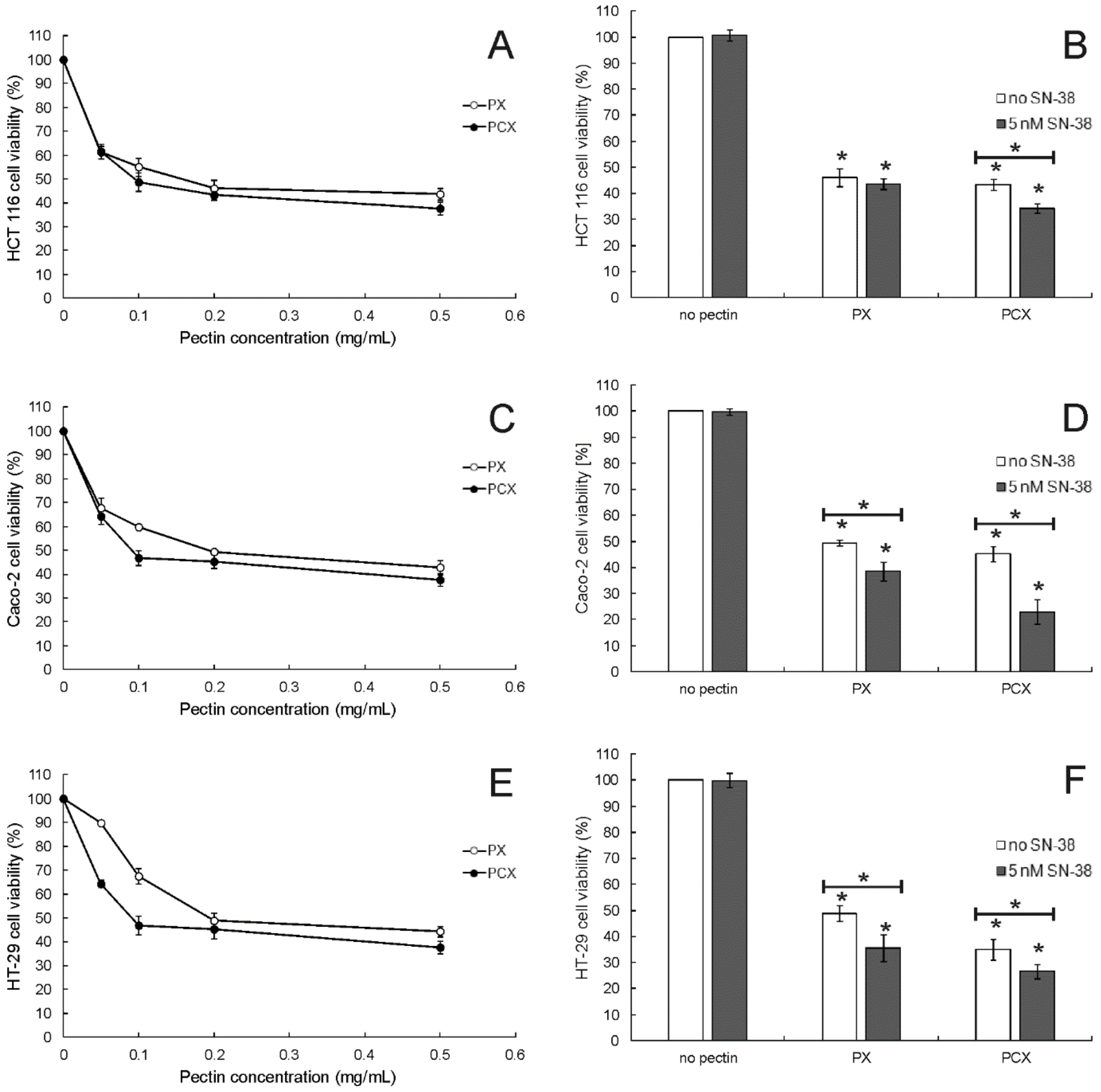

Figure S1. SRB cytotoxicity assay of pectins in HCT 116 (A), Caco-2 (C), HT-29 cells (E), and 0.2 mg/ml pectins in combination with SN-38 (B, D and F for HCT 116, Caco-2, and HT-29 cells, respectively). Incubation time was 48 h. The means of three experiments ± SD are presented (* $p < 0.05$). Statistical significance was checked between the studied probes and controls (no pectin) and between probes containing only pectin and pectin combined with SN-38.

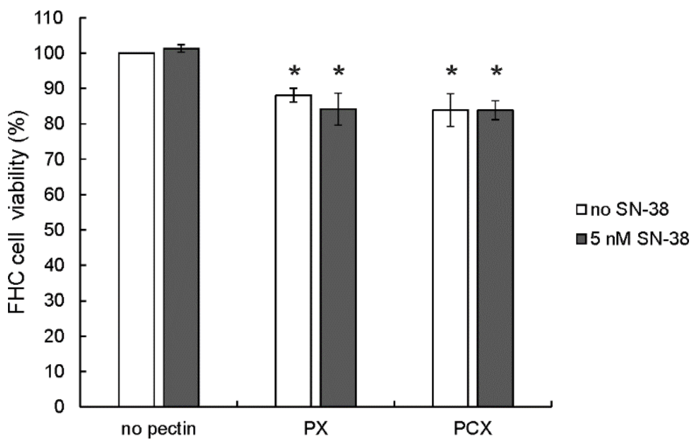

Figure S2. MTT cytotoxicity assay of pectins at 0.2 mg/mL in FHC cells. Incubation time was 48 h.
The means of three experiments ± SD are presented (* $p < 0.05$). Statistical significance was checked between the studied probes and controls (no pectin) and between probes containing only pectin and pectin combined with SN-38.

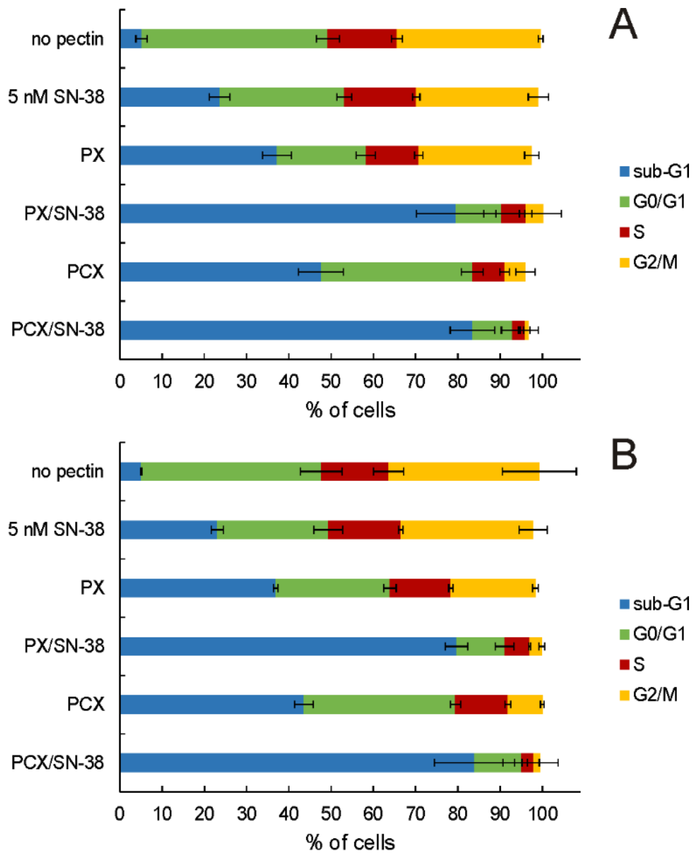

Figure S3. Cell cycle-dependent DNA content in **HCT 116 (A) and Caco-2 (B),** cells treated with 0.2 mg/ml of pectins and/or SN-38 for 48 hours. SubG1 population – dead cells, G0/G1 – mononuclear cells, S – DNA replication, G2/M – mitosis. The means of three experiments ± SD are presented (* $p < 0.05$).

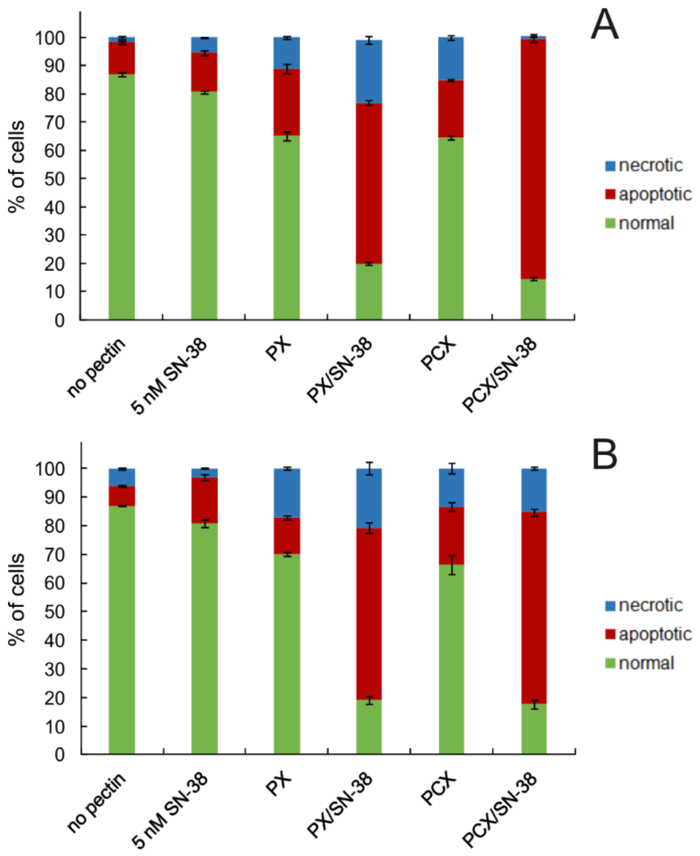

Figure S4. The proportion of normal, apoptotic , and necrotic cell populations as recorded by Annexin V apoptosis assay in HCT 116 (A) and Caco-2 (B) cells treated with pectins (0.2 mg/ml) and/or SN-38 for 48 hours. The means of three experiments ± SD are presented (* $p < 0.05$).

Cells were recognized as viable (Annexin-V and PI negative), apoptotic (Annexin-V positive and PI negative), and necrotic (Annexin-V and PI-positive) based on the measurement of cell-associated fluorescence of FITC-Annexin-V conjugate and PI.

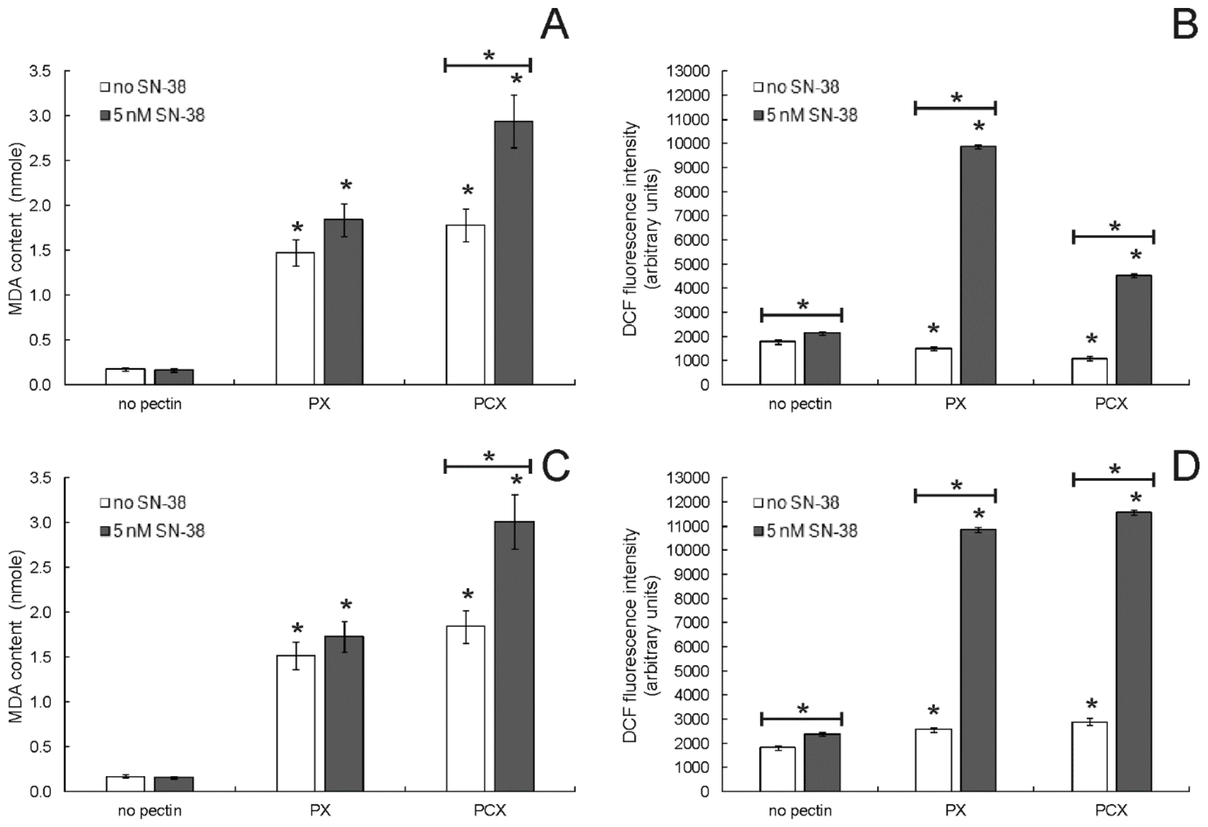

Figure S5. Lipid peroxidation (A and C) and ROS level (B and D) in HCT 116 (A and B) and Caco-2 (C and D) cells treated with 0.2 mg/ml pectins and/or 5 nM SN-38 for 48 hours.

The means of three experiments ± SD are presented (* $p < 0.05$). Statistical significance was checked between the studied probes and controls (no pectin) as well as between probes containing only pectin and pectin combined with SN-38.

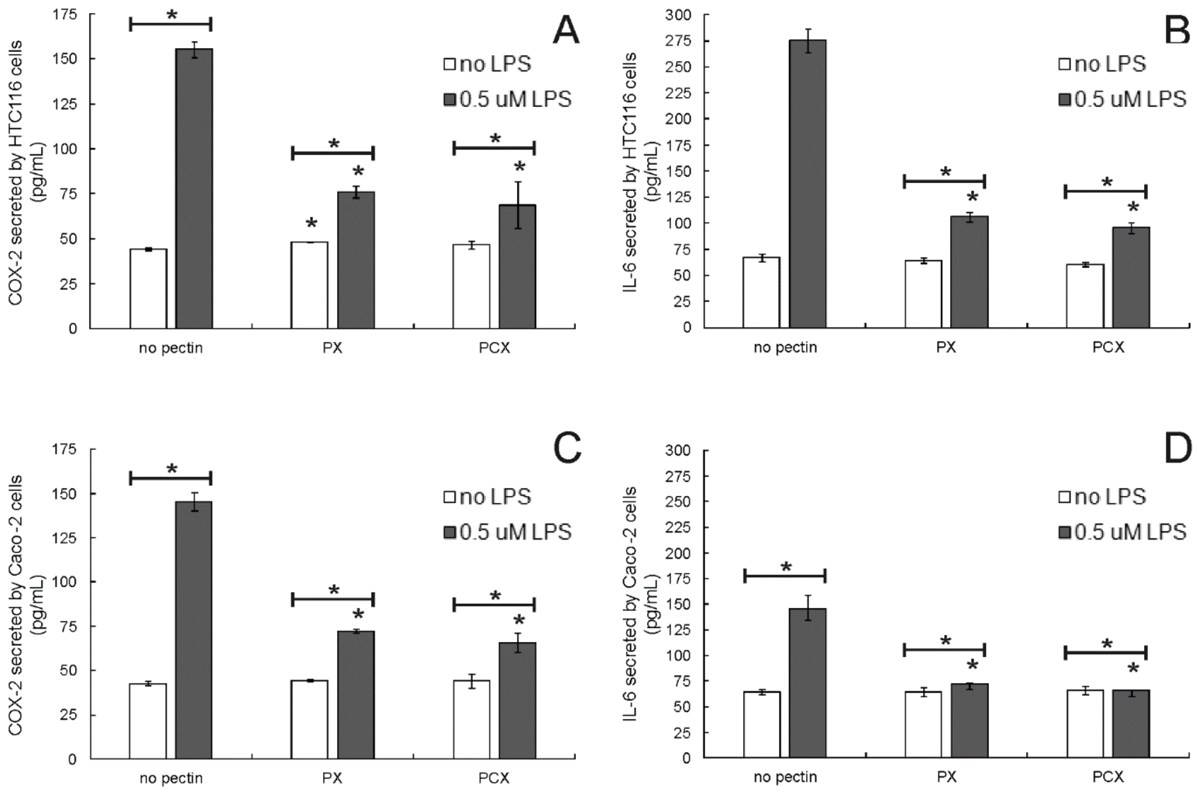

Figure S6. Amount of COX-2 (A and C) and IL-6 (B and D) in HCT 116 (A and B) and Caco-2 (C and D) cells treated with 0.2 mg/ml pectins and/or 0.5 μM LPS. Cells were pretreated with LPS for 24 hours and then incubated with pectins for 48 hours.

The means of three experiments ± SD are presented (* $p < 0.05$). Statistical significance was checked between the studied probes and controls (no pectin) as well as between probes containing only pectin and pectin combined with LPS.

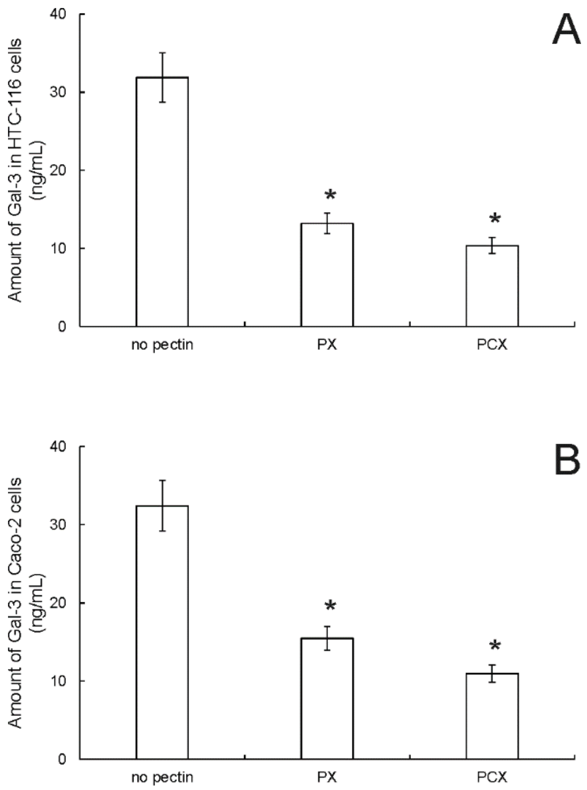

Figure S7. Amount of Gal-3 in HCT 116 (A) and Caco-2 (B) cells treated with 0.2 mg/ml pectins for 48 hours. The means of three experiments ± SD are presented (* $p < 0.05$). Statistical significance was checked between the studied probes and controls (no pectin).
